# Analysis of the Role of the Drought-Induced Gene *DRI15* and Salinity-Induced Gene *SI1* in *Alternanthera philoxeroides* Plasticity Using a Virus-Based Gene Silencing Tool

**DOI:** 10.3389/fpls.2017.01579

**Published:** 2017-09-12

**Authors:** Chao Bai, Peng Wang, Qiang Fan, Wei-Dong Fu, Le Wang, Zhen-Nan Zhang, Zhen Song, Guo-Liang Zhang, Jia-He Wu

**Affiliations:** ^1^The State Key Laboratory of Plant Genomics, Institute of Microbiology, Chinese Academy of Sciences Beijing, China; ^2^Institute of Environment and Sustainable Development in Agriculture, Chinese Academy of Agricultural Sciences Beijing, China

**Keywords:** *Alternanthera philoxeroides*, virus induced gene silencing (VIGS), drought stress, salinity stress, pgR107 VIGS vector

## Abstract

*Alternanthera philoxeroides* is a notoriously invasive weed that can readily adapt to different environmental conditions. Control of this weed is difficult, and it spreads easily and causes damage to native habitats and agriculture. In this study, our goal was to investigate the molecular mechanisms that lead to the ability of *A. philoxeroides* to invade new habitats, to adapt to environmental stresses, and to cause damage. We developed a simple and highly effective potato virus X-based virus-induced gene silencing (VIGS) approach. The VIGS approach was first used to silence the phytoene desaturase gene, which resulted in the expected photo-bleaching phenotype. Next, the VIGS approach was used to silence two additional genes, drought-induced protein gene 15 (*ApDRI15*) and salinity-induced protein gene 1 (*ApSI1*). When *ApDRI15* was knocked down, the plants were more sensitive to drought stress than the control plants, with smaller leaves, shorter internodes, and lower biomass. The *ApDRI15*-silenced plants had lower relative water content, lower free proline levels, and higher water loss rates than the control. Silencing of *ApSI1* significantly decreased tolerance to salinity, and the *ApSI1*-silenced plants were withered and smaller. These results indicate that the pgR107 VIGS approach is a simple and highly effective tool for dissecting gene function in *A. philoxeroides*. Further experiments with the VIGS approach will enhance our understanding of the molecular mechanisms of the adaptability and plasticity of *A. philoxeroides* and improve our ability to combat the damage caused by this weed.

## Introduction

*Alternanthera philoxeroides* (Max.), also called alligator weed, is a notoriously invasive weed. It originated in the Parana river region in southern America, but now has invaded the United States, Australia, New Zealand, China, and India (Sainty et al., 1998). This stoloniferous and amphibious weed grows in both terrestrial and aquatic conditions. *A. philoxeroides* can adapt to different habitats and to fluctuating environments (Wang et al., 2008, 2009; Dong et al., 2012; Fan et al., 2013). For instance, in arid land, its leaves are small, with short internodes and thin cavities, but in water the leaves are large, with long internodes and wide cavities (Wang et al., 2008; Dong et al., 2010). The plasticity and adaptability of *A. philoxeroides* facilitates invasion into new habitats, which damages agricultural production and ecological balance (Ye et al., 2003; You et al., 2016). *A. philoxeroides* has been become one of the most notoriously destructive weeds worldwide, but there are no effective measures to control this weed (Schooler et al., 2007). Therefore, there is a critical need for exploration of the underlying mechanisms involved in the plasticity and adaptability of *A. philoxeroides*.

Most of the major studies on *A. philoxeroides* have focused on plant morphology, ecology, taxonomy, and weed management (Sainty et al., 1998; Ye et al., 2003; Wang et al., 2005, 2008; Dong et al., 2010; Wu et al., 2016a,b). However, the underlying molecular mechanisms of invasion of this weed remain unclear, primarily because of a shortage of analytical techniques. Virus-induced gene silencing (VIGS) is a powerful approach in molecular biology and genetics, widely used to dissect gene function in plants. The modified VIGS vectors come from many plant RNA and DNA viruses, which are used in diverse dicot and monocot plants. For instance, there are VIGS vectors widely used in dicot plants, including tobacco rattle virus-derived vector (Ratcliff et al., 2001; Liu et al., 2002), bean pot mottle virus-derived vector (Zhang and Ghabrial, 2006), cabbage leaf curl virus-derived vector (Turnage et al., 2002), potato virus X vector (later modified pgR106/107 VIGS vector) (Faivre-Rampant et al., 2004), and tobacco mosaic rattle virus (George et al., 2010). In monocot plants, there are also several VIGS systems for analyzing the function of genes, such as barley stripe mosaic virus (Holzberg et al., 2002), brome mosaic virus (Ding et al., 2006), and bamboo mosaic virus (Liou et al., 2014). Very recently, cucumber mosaic virus-based VIGS in maize (Wang et al., 2016) and foxtail mosaic virus-based VIGS have been reported in barley, wheat, and foxtail millet (*Setaria italica*) (Liu et al., 2016). Thus, our goal was to develop a VIGS system for assessing gene function and for controlling *A. philoxeroides* invasion and damage.

In this study, we firstly explored several types of VIGS systems and successfully developed a pgR107 (PVX-based vector) VIGS approach in *A. philoxeroides*. First, we isolated a phytoene desaturase (*PDS*) gene from *A. philoxeroides* to use as a marker gene in the development of the VIGS system. Then, we employed this VIGS system to examine the role of the *A. philoxeroides* drought-induced protein gene 15 (*DRI15*, GenBank DQ985704.1) and the salinity-induced protein gene 1 (*SI1*, GenBank DQ489701) in response to stress from drought and high salinity. The pgR107 VIGS system is a simple and effective tool for analyzing *A. philoxeroides* gene function that will facilitate control of weed invasion and damage.

## Materials and Methods

### Plant Materials, Infiltration and Growth Conditions

*Alternanthera philoxeroides* was collected from field in Sichuan province in China, and cultured it in water supplemented with hoagland’s solution in growth chamber for asexual propagation. Then newly germinating internodes of branches were transferred to a pot for a single clone culture with many branches. The 3–4 internodes cut from different branches were inserted into cultural soil (two internodes above the ground) for propagation. When the two new internodes grew, the two opposite leaves at top internodes were used for VIGS infiltration. The new expanding leaves of inoculation plants were used for RNA and proteins extraction. All plants were grown in a growth chamber at 25°C with a 12-h light/12-h dark photoperiod cycle. The intensity of light is 2000 lum/sqf.

### PDS, DRI15, and SI1 cDNA Isolation and Plasmid Construction

pgR107 vector (Jones et al., 1999; Lu et al., 2003) was kindly given by Professor Zhendong Tian in Huazhong Agricultural University of China, was firstly applied to the asexual propagation plant of *A. philoxeroides* in this study. pgR107 vector is binary vector based on pGreen0000 backbone, in which CP promoter contains *Cal*I-*Smal*I-*Sal*I cloning sites.

**FIGURE 1:**
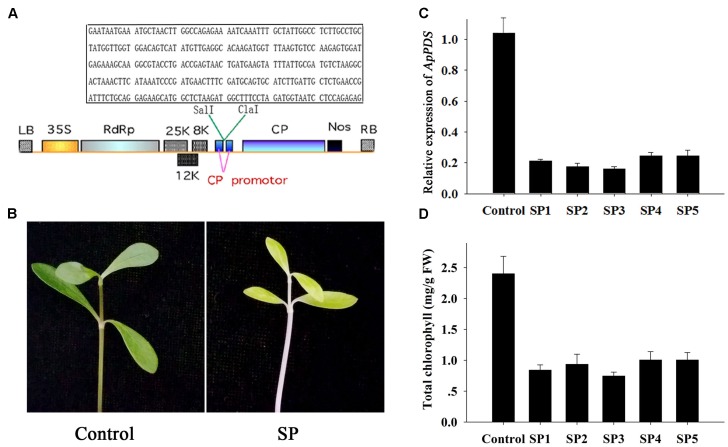
Development of *ApPDS*-silenced plants. **(A)** T-DNA schematic structure of the pgRPDS virus vector. LB and RB indicate the left and right T-DNA border sequences. 35S indicates the 35S promoter of cauliflower mosaic virus. RdRp indicates the potato virus X (PVX) 165K RNA-dependent RNA polymerase. 8K, 12K, and 25K indicate the PVX triple gene block movement proteins. CP indicates the viral coat protein gene. Nos indicates the nopaline synthase transcriptional terminator. The specific target sequence of the *ApPDS* gene is contained in the box. **(B)** The photo-bleaching phenotype. The control was plants agro-infiltrated with the empty vector. SP indicates the *ApPDS*-silenced plants, exhibiting photo-bleaching. **(C)** The expression level of *ApPDS*. SP1 through SP5 are five replicate *ApPDS*-silenced plants. **(D)** The total chlorophyll levels of new leaves from *PDS*-silenced plants. SP1 through SP5 are five *ApPDS*-silenced plants.

The *A. philoxeroides PDS* gene was amplified by PCR using DNA from *A. philoxeroides* leaves. The PCR primers were designed to amplify sequences conserved in other species of plants (Supplementary Table S1). The 936 base pair *PDS* fragment was amplified by PCR (**Supplementary Data Sheet S1**), and a 300 base pair specific fragment (**Figure 1A** in box) was inserted in the *Sal* I/*Cla* I sites of the pgR107 vector. The resulting vector was named pgRPDS (**Figure 1A**).

**FIGURE 2:**
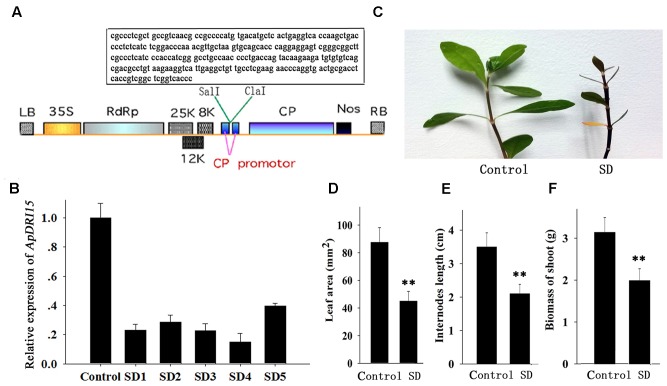
Sensitivity of *ApDRI15*-silenced plants to drought stress. **(A)** T-DNA schematic structure of pgRDRI15 virus vector. LB and RB indicate the left and right T-DNA border sequences. 35S indicates the 35S promoter of cauliflower mosaic virus. RdRp indicates the PVX 165K RNA-dependent RNA polymerase. 8K, 12K, and 25K indicate the PVX triple gene block movement proteins. CP indicates the viral coat protein gene. Nos indicates the nopaline synthase transcriptional terminator. The specific target sequence of the *ApDRI15* gene is displayed in the box. **(B)** The expression level of *ApDRI15*. The control is the plants agro-infiltrated with the empty vector. SD1 through SD5 are five replicate *ApDRI15*-silenced plants. Expression in the silenced plants was normalized to the expression in the control plant. **(C)** Drought-sensitive phenotype of the *ApDRI15*-silenced plants. SD is the *ApDRI15*-silenced plant. **(D)** The leaf area of the *ApDRI15*-silenced plants and the control. **(E)** The internode length of the *ApDRI15*-silenced plants and the control. **(F)** The shoot biomass of the *ApDRI15*-silenced plants and the control. Error bars represent the standard derivation of three biological replicated [*n* = 36 in **(D,E)**, *n* = 24 in **(F)**]. Double asterisks indicate statistically significant differences compared to the control, as determined using the Student’s *t-*test (*p* < 0.01).

The *ApDRI15* and *ApSI1* genes were first identified from a database of sequenced cDNAs prepared from *A. philoxeroides* leaves. After we blast two gene nucleotide sequences in NCBI, the specific sequence fragments in *A. philoxeroides* were selected for analyzing their functions. Then a special sequence fragment of *ApDRI15* (260 bp) or *ApSI1* (290 bp) was PCR-amplified with its specific primers (Supplementary Table S1). The *ApDRI15* and *ApSI1* PCR fragments were inserted in the sense orientation into the *Sal* I/*Cla* I sites of the pgR107 virus vector to create the pgRDRI15 and pgRDSI1 vectors, respectively (**Figures 2A**, **4A** in box).

### RNA Extraction and Analysis

Total RNA was extracted and purified from *A. philoxeroides* leaves using Plant Total RNA Isolation Kits (Sangon, Shanghai, China). One microgram of RNA was reverse-transcribed using the BluePrint 1st strand cDNA Synthesis Kit (Takara, Dalian, China) according to the manufacturer’s protocol.

The quantitative real-time PCR (qPCR) assay was conducted using the SYBR Premix Ex Taq kit (Takara) according to the manufacturer’s guidelines. All PCRs were run on an IQ5 multicolor detection system (Bio-Rad, Hercules, CA, United States). Gene expression was quantified using the ΔΔCt algorithm. To normalize gene expression, the universal *actin* gene was used as an internal standard. The gene-specific primers are listed in Supplementary Table S1. The experiment was repeated using three biological replicates.

### *Agrobacterium* Infiltration

The pgRPDS, pgRDRI15, and pgRSI vectors were introduced into *Agrobacterium tumefaciens* strain GV3101 with helper plasmid pJICSa_Rep (carries tetracycline as selection mark (5 μg/ml) was needed for replicating) by electroporation. Agrobacterial cells were grown, collected, and resuspended in MMA solution [10 mM MES (2-(*N*-Morpholino) ethanesulfonic acid), 10 mM MgCl_2_, 200 μM acetosyringone] to a final OD600 of 1.2. The *A. philoxeroides* shoots with 3–4 internodes (6–8 leaves) were used. The undersides of two fully expanded leaves were inoculated with *Agrobacterium* cells using a 1-mL needleless syringe. When another new 3–4 internodes grew after inoculation, they were cut and inserted a new pot with two internodes underground, which was regarded as silenced plants for salinity and drought treatment. Forty-five plants for each gene were repeated at least. Infiltrated plants were grown in a growth chamber at 25°C with a 12-h light/12-h dark photoperiod cycle.

### Transpirational Water Loss Assay

Transpirational water loss assay of detached leaves was conducted according to previous reports (Duan et al., 2012). Leaves of *ApDRI15*-silenced and control plants were detached and placed on an electronic balance for continuously weighting under room temperature condition. The reduction in fresh weight of the samples was presumed to be the result of water loss. This experiment was repeated three times.

### Relative Water Content

The relative water content of leaves was measured using the method of Parida et al. (2007). Fully expanded leaves were cut from the plants, and the fresh weight was recorded immediately. Then, the fresh portions were immersed in distilled water for 4 h and the turgid weight was recorded. Finally, the dry weight was recorded after drying for 48 h at 80°C in an oven. The relative water content was calculated according to the following formula:

Relative water content (%) = (Fresh weight - Dry weight)/(Turgid weight - Dry weight) × 100.

### Chlorophyll Content Assay

Chlorophyll content was measured using the method of Arnon (1949). Extracts were obtained from 0.1 g (fresh weight) leaf samples and were homogenized in 1 mL of 80% acetone to quantify the chlorophyll content via spectrophotometric analysis.

### Biomass Accumulation and Determination of Proline Content

Similar size of plants was used for each treatment. The total above-ground fresh weight biomass (including shoot and leaves) was measured immediately after harvesting after 15 days stress treatment and then the average biomass plant^-1^. The free proline content was measured using the method described by Bates et al. (1973). Leaf segments were homogenized in 3% sulfosalicylic acid, and the homogenates were centrifuged at 3000 × *g* for 20 min. Mixtures containing 2 mL of sample supernatant, 2 mL of acetic acid, and 2 mL of 2.5% acid ninhydrin solution were boiled for 30 min, and the absorbance at 520 nm (*A*_520_) was measured.

## Results

### Photo-Bleaching Phenotype in *ApPDS-*Silenced Plants

The *PDS* gene is widely used as a marker gene in many plant VIGS assays because the photo-bleaching phenotype is easy to visualize (Liu et al., 2002). Therefore, our first step was to isolate the *A. philoxeroides PDS* gene. Several nucleotide sequences of the *PDS* gene from diverse plants in the NCBI database were aligned (**Supplementary Figure S1**). We then designed PCR primers in highly conserved regions, as shown in Supplementary Table S1 and **Figure S1**. A 936 base-pair *PDS* fragment was obtained (**Supplementary Data Sheet S1**). A 300 base-pair specific fragment of the *PDS* gene (**Figure 1A** in box) was selected for gene silencing. The new leaves of *A. philoxeroides* plants were infiltrated with *Agrobacterium* cells containing pgRPDS or empty vector with a 1-mL syringe. Fourteen days after injection, the emerging leaves and internodes were photo-bleached in *PDS*-silenced plants presenting lighter green, while no changes were observed in plants infiltrated with empty vector (**Figure 1B**). The other pictures showing the similar lighter green phenotype were shown as **Supplementary Figure S2**. Additionally, the virus did not damage either these plants or the untreated plants. These data indicate that the pgR107 VIGS system can be used effectively and safely to dissect gene function in *A. philoxeroides*.

To analyze the effect of *PDS* silencing, qPCR was used to monitor the expression level of the gene in the agro-infiltrated plants. As shown **Figure 1C**, the *PDS* expression level was reduced in the new emerging leaves from five *PDS*-silenced plants. *PDS* was expressed at only 15–25% of the level expressed in plants injected with empty vector (the control). The chlorophyll contents of these treated plant leaves were tested to further confirm effective *PDS* knockdown. The total chlorophyll levels of new leaves from *PDS*-silenced plants were significantly lower than in leaves from the control (**Figure 1D**). The mean concentration of chlorophyll in *PDS*-silenced plants was only 35% of the control concentrations. The successful knockdown of the *PDS* gene indicates that the pgR107 VIGS system works well for dissection of *A. philoxeroides* gene function.

### VIGS Knockdown of *ApDRI15* Increased *A. philoxeroides* Susceptibility to Drought Stress

*Alternanthera philoxeroides* adapts readily to various abiotic stresses including drought and salinity. We isolated a drought response-related gene, named *ApDRI15*, which was submitted to the NCBI database. A sequence fragment of *ApDRI15* was inserted into the pgR107 virus vector to create the pgRDRI15 vector (**Figure 2A**). The *ApDRI15*-silenced plants were created by agro-infiltration with GV3101 containing pgRDRI15. The normal plant inoculating with pgR107 VIGS vector (empty vector) served as control. As shown in **Figure 2B**, the *ApDRI15* expression level in the five *ApDRI15*-silenced plants was only 15–40% of the expression level in the control leaves. The silenced plants with <25% of the control expression level were selected for further analysis with the drought-response assay.

**FIGURE 3:**
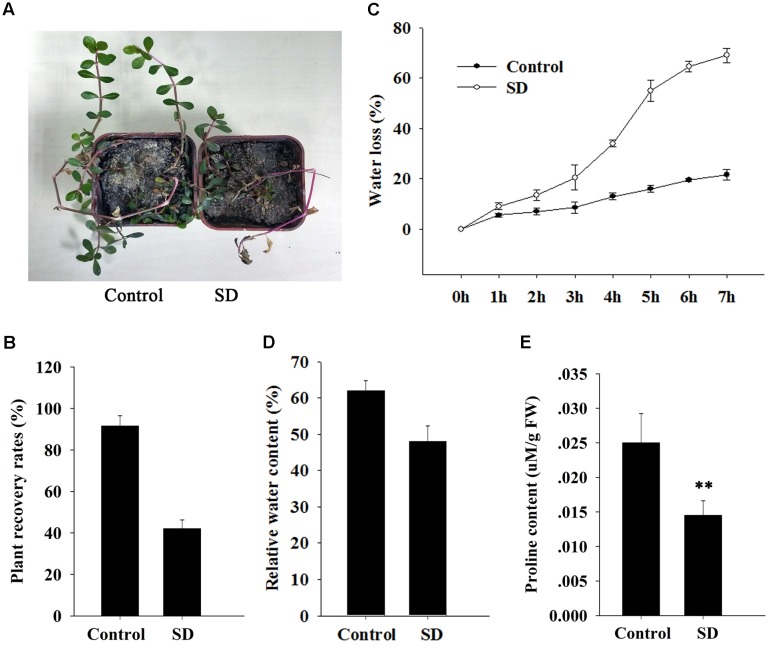
Effect of *ApDRI15* knockdown on survival and physiology of plants under drought stress. **(A)** Recovery of *ApDRI15*-silenced plants and control plants after re-watering. Control plants are plants agro-infiltrated with the empty vector. SD indicates *ApDRI15*-silenced plants. **(B)** Recovery rates of *ApDRI15* and control plants. **(C)** Water loss rates of detached leaves from *ApDRI15*-silenced plants and control plants. **(D)** Relative water contents of leaves of *ApDRI15*-silenced plants and control plants. **(E)** Free proline concentrations of *ApDRI15*-silenced plants and control plants. Error bars represent the standard derivation of three biological replicates [*n* = 92 in **(B)**, *n* = 30 in **(C,D)**, *n* = 15 in **(E)**]. Double asterisks indicate statistically significant differences compared to the control, as determined using the Student’s *t-*test (*p* < 0.01).

The *ApDRI15*-silenced plants and the control plants were grown in a greenhouse without watering. After 15 days (**Figure 2C**), the silenced plants were noticeably different from the controls. The silenced plants had significantly smaller leaves, shorter internodes, and less biomass than the controls (**Figures 2D–F**). After 21 days of drought, the silenced plants had wilted shoots and withered old leaves, evidence of greater drought sensitivity compared to the controls. Ten days after re-watering, 92% of control plants had recovered but only 42% of *ApDRI15*-silenced plants had recovered (**Figures 3A,B**).

We measured physiological parameters in order to evaluate the function of *ApDRI15* in the drought stress response. The detached leaf transpiration rate in the silenced plants was significantly higher than that in the controls (**Figure 3C**). The average leaf weight in the *ApDRI15*-silenced plants decreased by 70% after 8 h, whereas in the control plants the decrease was only 25%. The relative water content in leaves of silenced plants (48%) was lower than in leaves of control plants (62%) (**Figure 3D**). In addition, we monitored the level of free proline, an osmoprotective molecule that accumulates under drought stress, which has been reported as standard maker for plant drought resistance (Li et al., 2017). The average proline content of the *ApDRI15*-silenced plants was significantly lower than in the control plants (**Figure 3E**). These results suggested that the role of *ApDRI15* in drought stress resistance can be dissected using a pgR107 VIGS approach in *A. philoxeroides*.

### Knockdown of *ApSI1* Attenuated the Resistance of Plants to Salinity Stress

**FIGURE 4:**
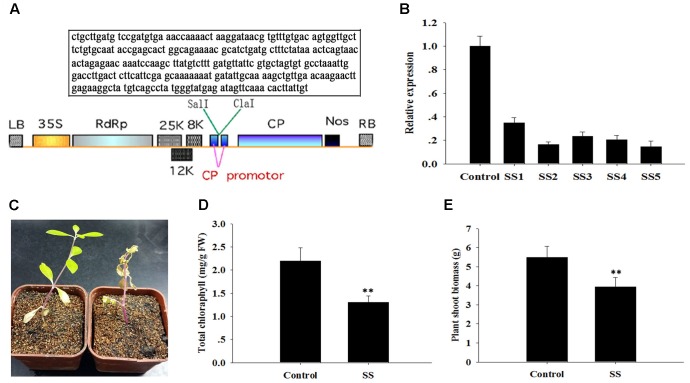
Sensitivity of *ApSI1*-silenced plants to salinity stress. **(A)** T-DNA schematic structure of pgRDSI1 virus vector. LB and RB indicate the left and right T-DNA border sequences. 35S indicates the 35S promoter of cauliflower mosaic virus. RdRp indicates the PVX 165K RNA-dependent RNA polymerase. 8K, 12K, and 25K indicate the PVX triple gene block movement proteins. CP indicates the viral coat protein gene. Nos indicates the nopaline synthase transcriptional terminator. The specific target sequence of *ApSI1* gene is displayed in the box. **(B)** The expression level of *ApsI1*. The control was plants agro-infiltrated with the empty vector. SS1 through SS5 are five replicate *ApSI1*-silenced plants. Expression in the *ApSI1*-silenced plants was normalized to the control. **(C)** Salinity-sensitive phenotype of *ApSI1*-silenced plants. SS indicates *ApSI1*-silenced plants. **(D)** Total chlorophyll contents of the *ApSI1*-silenced plants and control plants. **(E)** Shoot biomass of *ApSI1*-silenced plants and control plants. Error bars represent the standard derivation of three biological replicates (*n* = 24). Double asterisks indicate statistically significant differences between *ApSI1*-silenced and control plants, as determined using the Student’s *t-*test (*p* < 0.01).

We isolated another salinity inducible gene, *ApSI1*, that was deposited in NCBI database, and characterized its function with the pgR107 VIGS approach. As described above, the special fragment of *ApSI1* was cloned, and the resulting vector pgRSI was generated (**Figure 4A**). *ApSI1*-silenced plants and control plants were generated following agro-infiltration. The *ApSI1* expression levels in the silenced plants were significantly reduced, averaging only 22% of the control levels (**Figure 4B**). The *ApSI1*-silenced plants with low *ApSI1* expression levels (less than 22% of the control levels) were used to perform a salinity stress analysis. When silenced plants and control plants were treated with 300 μM sodium chloride, the *ApSI1*-silenced plant leaves were a paler shade of green than the control leaves. After 20 days of sodium chloride treatment, the *ApSI1*-silenced plants were withering, yet the control plants grew a bit yellowish under sodium chloride (**Figure 4C**). To evaluate the role of *ApSI1* in growth under salinity stress, we measured the total chlorophyll and the biomass of the silenced plants and the control plants 15 days after sodium chloride treatment. The average total chlorophyll level of *ApSI1*-silenced plant leaves was significantly lower than that of control leaves (**Figure 4D**). The fresh biomass of the silenced plants averaged 3.9 g per silenced plant shoot, but for the controls the value was 5.5 g (**Figure 4E**). The dissection of the role of ApSI1 in salinity tolerance further demonstrates that the pgR107 VIGS approach is an effective tool for analysis of gene function in *A. philoxeroides.*

## Discussion

*Alternanthera philoxeroides* is a notoriously invasive weed, and is difficult to prevent from its damages (Sainty et al., 1998; Pan et al., 2006; Fan et al., 2013). To date, the researches in the underlying molecular mechanisms of *A. philoxeroides*, exclude taxonomy and ecology and the integrated management is only limited to analyses of genome-wide DNA methylation and gene expression (Gao et al., 2010, 2015), due to insufficient molecular approaches. In this study, we successfully developed a simple and highly effective method in *A. philoxeroides*, the pgR107 VIGS system, using the *ApPDS* marker gene. We also used this VIGS approach to determine the roles of the *ApDRI15* and *ApSI1* genes in the *A. philoxeroides* response to drought and salinity stresses, respectively.

The disease symptoms of plants injected with the PVX vector are mild, which aids in the analysis of gene function (Faivre-Rampant et al., 2004; Jada et al., 2013; Ramanna et al., 2013). We used the pgR107 virus vector to silence *A. philoxeroides* genes, without obvious disease symptoms. Our results demonstrate that the PVX VIGS system (pgR107 vector) is extremely useful for dissecting gene function in *A. philoxeroides*, as demonstrated with the *ApPDS*, *ApDRI15*, and *ApSI1* genes. Additionally, because the *A. philoxeroides* line used in this study was developed through internode reproduction in a greenhouse for 2 years, further analysis of gene function in *A. philoxeroides* from other habitats is warranted because of the strong phenotypic plasticity of the species.

Virus-induced gene silencing is a valuable tool that can effectively silence individual genes or families of genes critical for plant development or resistance to biotic and abiotic stresses (George et al., 2015). This tool is a simple and rapid method for assessing gene function (Burch-Smith et al., 2004; Robertson, 2004; Becker and Lange, 2010; Cakir et al., 2010). *A. philoxeroides* can readily adapt to terrestrial and aquatic habitats. Moreover, the vegetative regeneration clones can exploit extremely diverse habitats, including dry lands, lakes, and high-salt areas, exhibiting notable morphological differences (Huai et al., 2003; Pan et al., 2006; Geng et al., 2007; Gao et al., 2010). Thus, this weed is able to colonize a wide range of habitats (Geng et al., 2006, 2007; Li and Ye, 2006; Pan et al., 2006; Wu et al., 2016a,b). In this study, we dissected the function of the drought-related gene *ApDRI15* and the salinity-related gene *ApSI1* using the VIGS method. *ApDRI15* knockdown significantly increased susceptibility of *A. philoxeroides* plants to drought stresses, resulting in lower biomass, smaller leaves, shorter internodes, higher water loss rates, lower relative water content, and lower proline level compared to the control plants. When the *ApSI1* gene was silenced in plants, the silenced plants withered, grew pale, and had lower biomass compared to the control plants. Therefore, the PVX VIGS system can be used to silence *A. philoxeroides* genes to determine their function, thereby elucidating the molecular mechanisms of invasion and colonization.

In this study, *A. philoxeroides DRI15* were isolated for analyzing its function against drought stress. In NCBI database, *A. philoxeroides DRI15* gene had been previously submitted, and annotated as drought-induced gene. We also further confirmed that the *DRI15* expression was response to drought stress by qPCR analysis (**Supplementary Figure S3**). However, *DRI15* has not been identified in other plants. The structural ortholog of *A. philoxeroides DRI15* putative protein is highly similar with ribosomal protein s3a and s1-a (identification of 93.1 and 91.8% at protein level, respectively, **Supplementary Data Sheet S2**). The ribosomal protein served as a multi-functional protein interacted with p53, which was associated with cellular stress in human (Goodin and Rutherford, 2002; Zhan, 2005; Yadavilli et al., 2009). Thereby, the function of *DRI15* in plants should be extensively explored in drought-tolerance.

We selected a salt-induced gene of *A. philoxeroides*, *SI1*, for evaluating its function by VIGS method. Up-to-now, there were many researches involved in salt-induced genes. For instance, *Salicornia brachiata SI-1* and *SI-2* genes were overexpressed in tobacco, conferring salinity tolerance (Yadav et al., 2014; Kumari et al., 2017). A salt-responsive gene in wheat, *TaDi19A*, was identified to play a vital role in the plant salt-tolerance (Li et al., 2010). In the present study, *ApSI1* played important roles in *A. philoxeroides* plant salt-tolerance according to the results of silenced plants under salt treatment.

## Conclusion

We have successfully developed a pgR107 VIGS approach in *A. philoxeroides*. By dissecting the role of the *ApDRI15* and *ApSI1* genes in the response of plants to the stress of drought and salinity, we have demonstrated that the PVX VIGS system is a simple and rapid method for assessing the role of individual genes and gene families in *A. philoxeroides*. This tool will be invaluable for revealing the potential molecular mechanisms of *A. philoxeroides* invasion and colonization and for developing measures to check weed invasion, control damage to agriculture, and protect ecological balance in invaded habits.

## Author Contributions

J-HW and G-LZ designed the experimental strategy, analyzed the data, and drafted the manuscript. CB and W-DF edited the manuscript. CB and PW cloned the *A. philoxeroides* PDS gene, created the *ApDRI15* and *ApSI1* constructs, and set up the pgR107 VIGS system. QF measured the water content. LW conducted the transpirational water loss assay. Z-NZ carried out the chlorophyll content assay. ZS measured and calculated the biomass. All authors read and approved the final manuscript.

## Conflict of Interest Statement

The authors declare that the research was conducted in the absence of any commercial or financial relationships that could be construed as a potential conflict of interest.

## Supplementary Material

FIGURE S1 Alignment of several nucleotide sequences of the *PDS* gene from diverse plants in the NCBI database.

FIGURE S2 The photo-bleaching phenotype. The control was plants agro-infiltrated with the empty vector in **(A–E)** indicate the *ApPDS*-silenced plants, exhibiting photo-bleaching.

FIGURE S3 The relative expression level of *ApDRI15* in normal plants under the control (normal-watering) and drought treatment. Error bars represent the standard derivation of three biological replicates (*n* = 45).

DATA SHEET S1 The partial sequence of *ApPDS* including 936bp.

DATA SHEET S2 The alignment of structural ortholog of *Alternanthera philoxeroides DRI15* putative protein in Phyre^2^ database.
