# Supplementary figures and images for: Analysis of the Role of the Drought-Induced Gene DRI15 and Salinity-Induced Gene SI1 in Alternanthera philoxeroides Plasticity Using a Virus-Based Gene Silencing Tool
Source: Front Plant Sci. 2017 Sep 12;8:1579. doi: 10.3389/fpls.2017.01579 (PMC5601067; doi:10.3389/fpls.2017.01579)

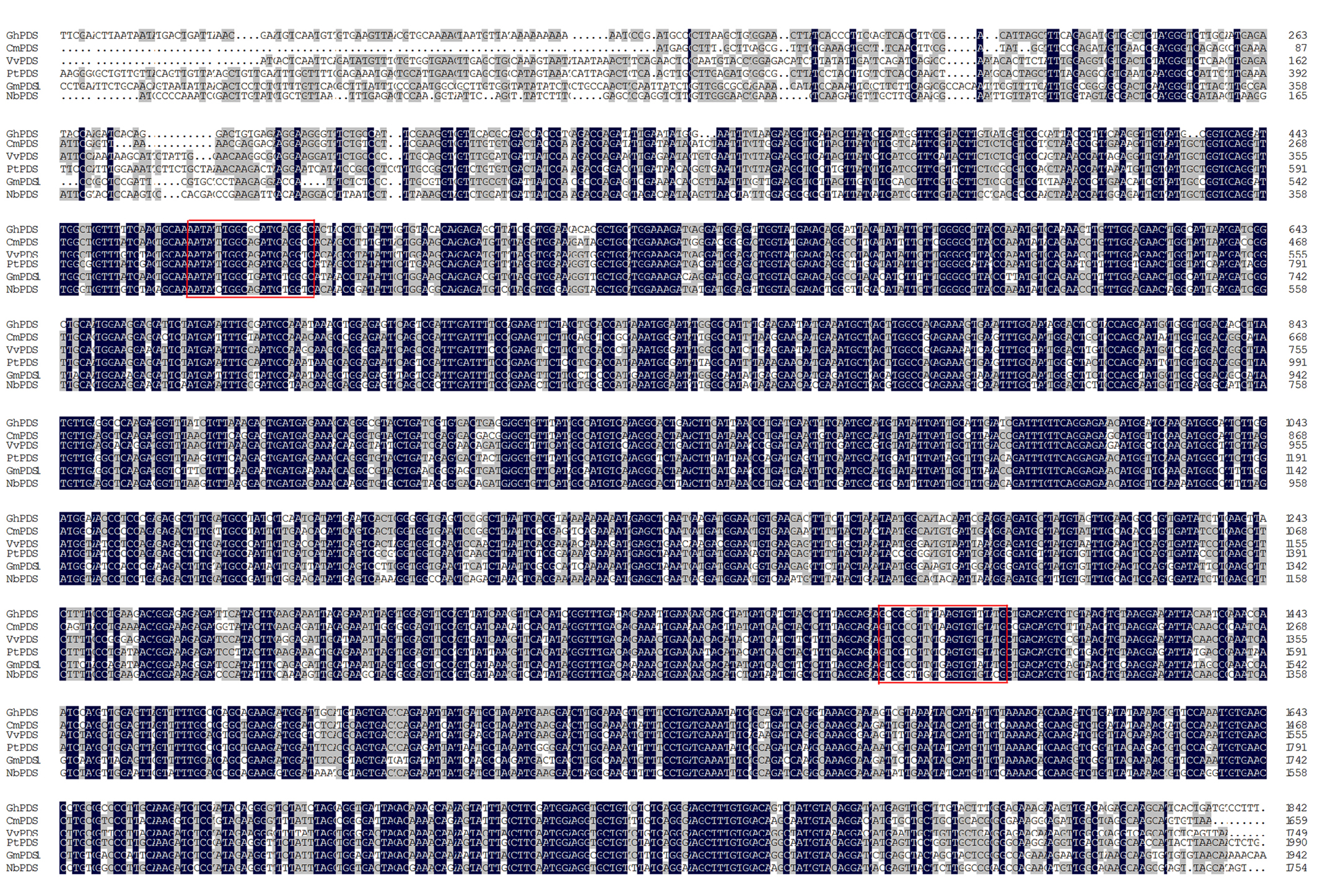

Supplement: FIGURE S1 — Alignment of several nucleotide sequences of the PDS gene from diverse plants in the NCBI database. [file Image_1.JPEG]

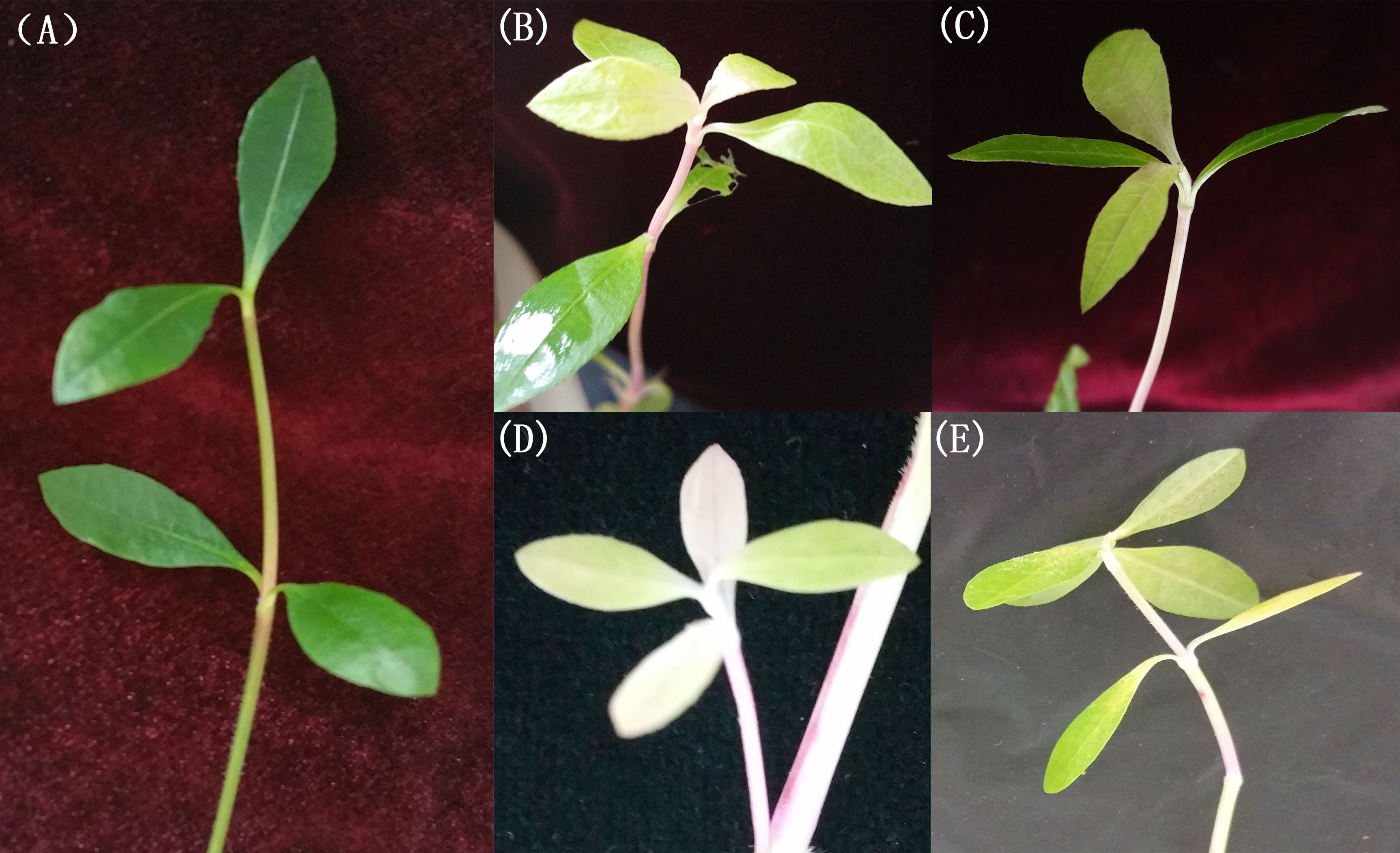

Supplement: FIGURE S2 — The photo-bleaching phenotype. The control was plants agro-infiltrated with the empty vector in (A–E) indicate the ApPDS-silenced plants, exhibiting photo-bleaching. [file Image_2.JPEG]

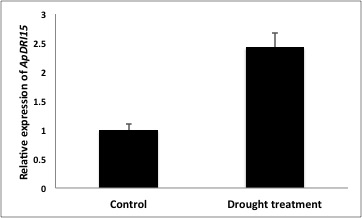

Supplement: FIGURE S3 — The relative expression level of ApDRI15 in normal plants under the control (normal-watering) and drought treatment. Error bars represent the standard derivation of three biological replicates (n = 45). [file Image_3.JPEG]
