# Supplementary material for: Analysis of the Role of the Drought-Induced Gene DRI15 and Salinity-Induced Gene SI1 in Alternanthera philoxeroides Plasticity Using a Virus-Based Gene Silencing Tool
Source: Front Plant Sci. 2017 Sep 12;8:1579. doi: 10.3389/fpls.2017.01579 (PMC5601067; doi:10.3389/fpls.2017.01579)
Supplement: DATA SHEET S1 — The partial sequence of ApPDS including 936bp. [file Data_Sheet_1.DOCX]

**Data S1. The partial sequence of *ApPDS* including 936bp.**

1 GACTATTTTA GCAGATGCAG GCCACAAGCC CATCTTGCTG GAAGCACGTG ATGTTTTAGG

61 TGGAAAGGTG GCAGCATGGA AAGATGAGGA TGGTGACTGG TACGAGACTG ATTACATATA

121 TTTTTTGGGG CATACCCTAA TGTGCAGAAT CTATTTGGGG AGCTTGGTAT TAACGACCGT

181 TTGCAATGGA AGGAGCACTC TATGATTTTT GCAATGCCTA GCAAGCCTGG AGAGTTTAGT

241 CGATTTGATT TTGCTGAAGT TCTGCCTGCA CCAATAAATG GCATTTGGGC AATCTTAAAG

301 GAATAATGAA ATGCTAACTT GGCCAGAGAA AATCAAATTT GCTATTGGCC TCTTGCCTGC

361 TATGGTTGGT GGACAGTCAT ATGTTGAGGC ACAAGATGGT TTAAGTGTCC AAGAGTGGAT

421 GAGAAAGCAA GGCGTACCTG ACCGAGTAAC TGATGAAGTA TTTATTGCGA TGTCTAAGGC

481 ACTAAACTTC ATAAATCCCG ATGAACTTTC GATGCAGTGC ATCTTGATTG CTCTGAACCG

541 ATTTCTGCAG GAGAAGCATG GCTCTAAGAT GGCTTTCCTA GATGGTAATC CTCCAGAGAG

601 TTGTGCATGC CTATTGTTCA GCATATTGAG TCTCTTGGTG GTGAAGTGCG TTTGAATTCG

661 CGTATCAAAA AAATTGAGTT AAATCAGGAT GGAAGCGTGA AGAATTTTTT GCTAAGTAAT

721 GGAAGGGAGA TAAGAGGGGA TGCTTATGTC TTTGCCGCTC CAGTTGACAT CCTGAAATTG

781 CTTCTGCCTG ATGATTGGAA AGAAATCCCA TACTTCAAAA AATTGGAGAA GTTAGTAGGA

841 GTGCCGGTGA TTAATGTTCA CATATGGTTT GATAGGAAAT TAAAGAATAC ATATGATCAT

901 CTACTCTTCA GCAGGAGTTC TCTTCTGGTG TGTGTG
