# Supplementary material for: Analysis of the Role of the Drought-Induced Gene DRI15 and Salinity-Induced Gene SI1 in Alternanthera philoxeroides Plasticity Using a Virus-Based Gene Silencing Tool
Source: Front Plant Sci. 2017 Sep 12;8:1579. doi: 10.3389/fpls.2017.01579 (PMC5601067; doi:10.3389/fpls.2017.01579)
Supplement: DATA SHEET S2 — The alignment of structural ortholog of Alternanthera philoxeroides DRI15 putative protein in Phyre2 database. [file Data_Sheet_2.PDF]

Phyre2

Email	baichao37@126.com
Description	Undefined
Date	Mon Jul 17 03:06:44 BST 2017
Unique Job ID	c0651bb52132370e

Detailed template information

#	Template	Alignment Coverage	3D Model	Confidence	% i.d.	Template Information
1	c3bk6C_	Alignment	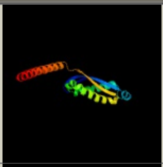	99.9	26	**PDB header:**membrane protein<br>**Chain:** C: **PDB Molecule:**ph stomatin;<br>**PDBTitle:** crystal structure of a core domain of stomatin from2 pyrococcus horikoshii
2	d1wina_	Alignment	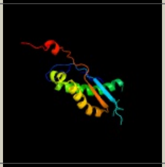	99.8	20	**Fold:**EF-Ts domain-like<br>**Superfamily:**Band 7/SPFH domain<br>**Family:**Band 7/SPFH domain
3	c4fvjB_	Alignment	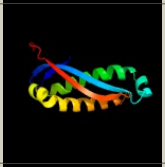	99.8	23	**PDB header:**membrane protein<br>**Chain:** B: **PDB Molecule:**stomatin;<br>**PDBTitle:** spfh domain of the mouse stomatin (crystal form 2)
4	c2rpbA_	Alignment	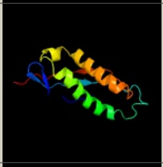	99.8	30	**PDB header:**membrane protein<br>**Chain:** A: **PDB Molecule:**hypothetical membrane protein;<br>**PDBTitle:** the solution structure of membrane protein
5	c2zv4O_	Alignment	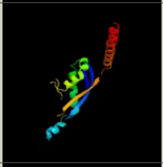	97.8	14	**PDB header:**structural protein<br>**Chain:** O: **PDB Molecule:**major vault protein;<br>**PDBTitle:** the structure of rat liver vault at 3.5 angstrom resolution
6	c2qzvB_	Alignment	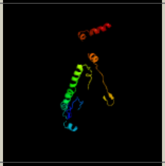	95.6	16	**PDB header:**structural protein<br>**Chain:** B: **PDB Molecule:**major vault protein;<br>**PDBTitle:** draft crystal structure of the vault shell at 9 angstroms2 resolution
7	c3zey0_	Alignment	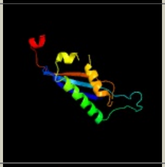	93.1	15	**PDB header:**ribosome<br>**Chain:** 0: **PDB Molecule:**40s ribosomal protein s3a, putative;<br>**PDBTitle:** high-resolution cryo-electron microscopy structure of the trypanosoma2 brucei ribosome
8	c3j3aB_	Alignment	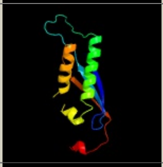	93.0	13	**PDB header:**ribosome<br>**Chain:** B: **PDB Molecule:**40s ribosomal protein s3a;<br>**PDBTitle:** structure of the human 40s ribosomal proteins
9	c3u5gB_	Alignment	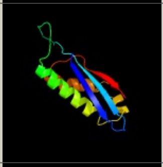	91.8	16	**PDB header:**ribosome<br>**Chain:** B: **PDB Molecule:**40s ribosomal protein s1-a;<br>**PDBTitle:** the structure of the eukaryotic ribosome at 3.0 a resolution. this2 entry contains proteins of the 40s subunit, ribosome b
10	c2xzm4_	Alignment	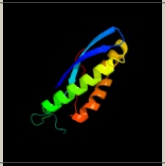	87.8	18	**PDB header:**ribosome<br>**Chain:** 4: **PDB Molecule:**40s ribosomal protein s3a;<br>**PDBTitle:** crystal structure of the eukaryotic 40s ribosomal2 subunit in complex with initiation factor 1. this file3 contains the 40s subunit and initiation factor for4 molecule 1
11	c3j20A_	Alignment	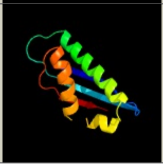	81.6	17	**PDB header:**ribosome<br>**Chain:** A: **PDB Molecule:**30s ribosomal protein s3ae;<br>**PDBTitle:** promiscuous behavior of proteins in archaeal ribosomes revealed by2 cryo-em: implications for evolution of eukaryotic ribosomes (30s3 ribosomal subunit)

12	d1omha_	Alignment	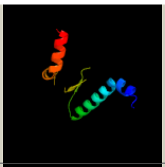	30.3	16	**Fold:**Origin of replication-binding domain, RBD-like<br>**Superfamily:**Origin of replication-binding domain, RBD-like<br>**Family:**Relaxase domain
13	c3lhkA_	Alignment	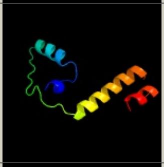	10.7	15	**PDB header:**dna binding protein<br>**Chain:** A: **PDB Molecule:**putative dna binding protein mj0014;<br>**PDBTitle:** crystal structure of putative dna binding protein from2 methanocaldococcus jannaschii.
14	d5ruba2	Alignment	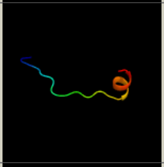	9.5	5	**Fold:**Ferredoxin-like<br>**Superfamily:**RuBisCO, large subunit, small (N-terminal) domain<br>**Family:**Ribulose 1,5-bisphosphate carboxylase-oxygenase
15	d1gk8a2	Alignment	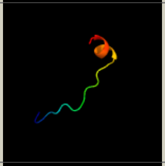	9.2	5	**Fold:**Ferredoxin-like<br>**Superfamily:**RuBisCO, large subunit, small (N-terminal) domain<br>**Family:**Ribulose 1,5-bisphosphate carboxylase-oxygenase
16	d2hsga1	Alignment	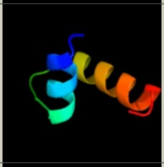	8.9	9	**Fold:**lambda repressor-like DNA-binding domains<br>**Superfamily:**lambda repressor-like DNA-binding domains<br>**Family:**GalR/LacI-like bacterial regulator
17	d1bwva2	Alignment	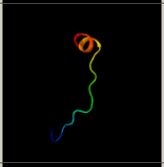	8.8	5	**Fold:**Ferredoxin-like<br>**Superfamily:**RuBisCO, large subunit, small (N-terminal) domain<br>**Family:**Ribulose 1,5-bisphosphate carboxylase-oxygenase
18	d2d69a2	Alignment	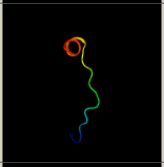	8.7	16	**Fold:**Ferredoxin-like<br>**Superfamily:**RuBisCO, large subunit, small (N-terminal) domain<br>**Family:**Ribulose 1,5-bisphosphate carboxylase-oxygenase
19	d1lcda_	Alignment	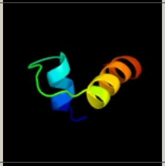	7.3	18	**Fold:**lambda repressor-like DNA-binding domains<br>**Superfamily:**lambda repressor-like DNA-binding domains<br>**Family:**GalR/LacI-like bacterial regulator
20	d2ha9a1	Alignment	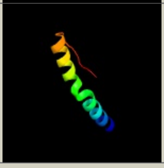	7.2	11	**Fold:**PFL-like glycyl radical enzymes<br>**Superfamily:**PFL-like glycyl radical enzymes<br>**Family:**SP0239-like
21	d1wdda2	Alignment	not modelled	7.0	5	**Fold:**Ferredoxin-like<br>**Superfamily:**RuBisCO, large subunit, small (N-terminal) domain<br>**Family:**Ribulose 1,5-bisphosphate carboxylase-oxygenase
22	d1ykwa2	Alignment	not modelled	6.7	11	**Fold:**Ferredoxin-like<br>**Superfamily:**RuBisCO, large subunit, small (N-terminal) domain<br>**Family:**Ribulose 1,5-bisphosphate carboxylase-oxygenase
23	d2bjca1	Alignment	not modelled	6.7	18	**Fold:**lambda repressor-like DNA-binding domains<br>**Superfamily:**lambda repressor-like DNA-binding domains<br>**Family:**GalR/LacI-like bacterial regulator
24	d1qd1a2	Alignment	not modelled	6.6	17	**Fold:**Ferredoxin-like<br>**Superfamily:**Formiminotransferase domain of formiminotransferase-cyclodeaminase.<br>**Family:**Formiminotransferase domain of formiminotransferase-cyclodeaminase.
25	d1geha2	Alignment	not modelled	6.5	11	**Fold:**Ferredoxin-like<br>**Superfamily:**RuBisCO, large subunit, small (N-terminal) domain<br>**Family:**Ribulose 1,5-bisphosphate carboxylase-oxygenase
26	c3r7wC_	Alignment	not modelled	6.3	15	**PDB header:**protein transport<br>**Chain:** C: **PDB Molecule:**gtp-binding protein gtr1;<br>**PDBTitle:** crystal structure of gtr1p-gtr2p complex
27	c3s5pA_	Alignment	not modelled	6.0	20	**PDB header:**isomerase<br>**Chain:** A: **PDB Molecule:**ribose 5-phosphate isomerase;<br>**PDBTitle:** crystal structure of ribose-5-phosphate isomerase b rpib from giardia2 lamblia
28	d1bxna2	Alignment	not modelled	5.7	18	**Fold:**Ferredoxin-like<br>**Superfamily:**RuBisCO, large subunit, small (N-terminal) domain<br>**Family:**Ribulose 1,5-bisphosphate carboxylase-oxygenase
						**Fold:**lambda repressor-like DNA-binding domains

29	d1qpza1	Alignment	not modelled	5.6	12	**Superfamily:**lambda repressor-like DNA-binding domains **Family:**GalR/LacI-like bacterial regulator
30	c2lcvA_	Alignment	not modelled	5.6	21	**PDB header:**transcription regulator **Chain:** A: **PDB Molecule:**hth-type transcriptional repressor cytr; **PDBTitle:** structure of the cytidine repressor dna-binding domain; an alternate2 calculation
31	d1svda2	Alignment	not modelled	5.3	6	**Fold:**Ferredoxin-like **Superfamily:**RuBisCO, large subunit, small (N-terminal) domain **Family:**Ribulose 1,5-bisphosphate carboxylase-oxygenase
32	d1efaa1	Alignment	not modelled	5.3	18	**Fold:**lambda repressor-like DNA-binding domains **Superfamily:**lambda repressor-like DNA-binding domains **Family:**GalR/LacI-like bacterial regulator
33	d8ruca2	Alignment	not modelled	5.3	12	**Fold:**Ferredoxin-like **Superfamily:**RuBisCO, large subunit, small (N-terminal) domain **Family:**Ribulose 1,5-bisphosphate carboxylase-oxygenase
34	d1rbla2	Alignment	not modelled	5.2	24	**Fold:**Ferredoxin-like **Superfamily:**RuBisCO, large subunit, small (N-terminal) domain **Family:**Ribulose 1,5-bisphosphate carboxylase-oxygenase
