# Supplementary material for: Analysis of the Role of the Drought-Induced Gene DRI15 and Salinity-Induced Gene SI1 in Alternanthera philoxeroides Plasticity Using a Virus-Based Gene Silencing Tool
Source: Front Plant Sci. 2017 Sep 12;8:1579. doi: 10.3389/fpls.2017.01579 (PMC5601067; doi:10.3389/fpls.2017.01579)
Supplement: Supplementary file 6 [file Table_1.DOCX]

| Gene or fragment names | Primer | Experiments |
| --- | --- | --- |
| *ApPDS* | F: attgctggtggaggtttggctgg  R: gacatgtcagcatacacact | Gene clone |
|  |  |  |
| *ApPDS* fragment | F: gaataatgaaatgctaacttg  R: ctctctggaggattaccatc | VIGS |
| *ApDRI15* | F: cgccctcgctgccgtcaac  R:gggtgaccgagccgacgg | VIGS and qPCR |
| *ApSI1* | F: ctgcttgatgtccgatgtg  R:acaataagtgtttgaactat | VIGS and qPCR |
| *Actin* | F:actcagtattgtaaaagatggcc  R: agcatcatcaccagcaaaac | qPCR |

**Table S1. The primers used in experiments.**
